# Surface Wave Diffraction Pattern Recorded on AlpArray: Cameroon Volcanic Line Case Study

**DOI:** 10.1029/2019JB019102

**Published:** 2020-07-21

**Authors:** Petr Kolínský, Felix M. Schneider, Götz Bokelmann

**Affiliations:** ^1^ Department of Meteorology and Geophysics University of Vienna Vienna Austria; ^2^ Section “Seismology” Helmholtz Centre Potsdam—German Research Centre for Geosciences (GFZ) Potsdam Germany

**Keywords:** surface waves, diffraction, Cameroon volcanic line, hotspot, AlpArray, tomography

## Abstract

Stripe‐like patterns of surface wave arrival angle deviations have been observed by several seismological studies around the world, but this phenomenon has not been explained so far. Here we test the hypothesis that systematic arrival angle deviations observed at the AlpArray broadband seismic network in Europe are interference patterns caused by diffraction of surface waves at single small‐scaled velocity anomalies. We use the observed pattern of Rayleigh waves from two earthquakes under the Southern Atlantic Ocean, and we fit this pattern with theoretical arrival angles derived by a simple modeling approach describing the interaction of a seismic wavefield with small anomalies. A grid search inversion scheme is implemented, which indicates that the anomaly is located in Central Africa, with its head under Cameroon. Moreover, the inversion enables the characterization of the anomaly: The anomaly is inferred to be between 320 and 420 km wide, matching in length the 2,500 km long upper mantle low‐velocity region under the volcano‐capped swells of the Cameroon volcanic line. We show that this approach can be generally used for studying the upper mantle anomalies worldwide.

## Key Points

- Arrival angle deviations of surface waves are explained by interference of diffracted wavefronts after passing a distant anomaly
- We find a strong, elongated low‐velocity region in the upper mantle under the Cameroon volcanic line
- These findings can help for understanding the nature of small‐scale convection in the upper mantle

## 1 Introduction

Peculiar patterns of amplitudes and arrival angle deviations of surface waves had been observed by Pollitz (2008) (“band‐like patterns” of amplitudes), Liang and Langston (2009) (“belts of negative and positive azimuth variations”), Lin et al. (2012) (“striping pattern” of amplitudes), Foster et al. (2014) (“banded appearance,” “banded pattern” of arrival angle deviations) and Liu and Holt (2015) (“banding pattern” of various gradiometry parameters) for the USArray data, and by Chen et al. (2018) (“belt‐like pattern” of arrival angle anomalies) for NECESSArray (Northeast China). Even though all these studies gave hints on the causes of these observations, a general explanation is missing. Recently, similar stripe‐like spatial patterns were observed for the AlpArray data by Kolínský and Bokelmann (2019). The latter study hypothesized that these stripe‐like arrival angle deviations could be caused by a diffraction after the wavefield has passed a single small‐scale scatterer. This suggestion was based mainly on the observed lateral shift of the stripes with period.

In our current study, we address this observation and hypothesis. Using the modeling approach by Nolet and Dahlen (2000) (referred as N&D2000 from now on), we show that interference of waves diffracted at a single anomaly can indeed cause stripe‐like arrival angle patterns as the ones observed.

Arrival angle deviations, in general, had already been observed locally before data from regional networks have become available. The deviations had mostly been attributed to off‐great‐circle propagation, multipathing, to diffraction and scattering along the raypaths, or to diffraction and scattering near to the source (see Kolínský and Bokelmann, 2019 and references therein). However, localized observations using small‐aperture arrays (e.g., Cotte et al., 2000) could not reveal the stripe‐like appearance of the observables as the size of the stripes exceeded the size of observations. The stripe‐like (banded) observations at large regional networks (USArray, NECESSArray, AlpArray) show that these earlier suggestions cannot serve as a general explanation of these patterns. We apparently observe finite‐frequency effects of the wavefield interacting with small‐scale anomalies. In our study, we are interested in arrival angle observations, the directional part of the phase velocity (slowness vector). We will study phase velocities and other (gradiometry) observables in a subsequent paper.

This paper tests the hypothesis that the observed arrival angle deviations of Rayleigh waves propagating from two neighboring earthquakes in the South Atlantic Ocean (presented in Kolínský and Bokelmann, 2019) can be explained by the interaction of the surface wavefield with a single anomaly that is located off the great circle path connecting the source and receivers. We address the question of which feature causes the diffraction. We locate and characterize it. We use a simple modeling approach, and we invert the arrival angle deviations observed at the AlpArray. The found feature corresponds to a known upper mantle anomaly under Central Africa, which is associated with the Cameroon volcanic line (CVL). The shape of the anomaly, its strength, and its position as derived from our new method can possibly allow us to discuss different models that have been proposed as causes of the anomaly.

Diffracted body waves have been used recently to study small‐scale variations in the deep mantle (Cottaar & Romanowicz, 2012; Yuan & Romanowicz, 2017). This has shown that it is in principle possible to resolve small‐scale features at scales of a few hundred kilometers by studying diffraction effects; in these cases, the root zones of the Hawaiian and Iceland plumes in the deepest part of the mantle were imaged successfully. In our current study, we show that diffraction pattern of surface waves can be used to study the anomalies in the upper mantle.

Global tomography using surface waves has recently reached a resolution in the upper mantle of a few hundred kilometers, and images of small‐scale convection have begun to emerge from such studies (Debayle et al., 2016 and references therein). Regional studies achieve even better resolution (e.g., Adams et al., 2015). The latter paper has focused on the structure of the CVL; however, imaging was possible only onshore, where the stations were deployed. Our technique enables resolving the upper mantle anomalies with a resolution comparable with the regional experiments but from a distance, which allows for studying the structure under the oceans or, as in the case of the CVL, under both the oceanic and the continental lithosphere. It is important to resolve such smaller‐scale variation of mantle properties to better understand mantle dynamics. Efforts have been made to use full waveform inversion (e.g., French et al., 2013) for that purpose. Such techniques can use more information than what is available in the arrival times, for example, amplitudes of waves, but also waveform shapes and diffraction effects. Our approach follows this goal, allowing for sharp imaging of upper mantle anomalies. The CVL is shown here as an example. In the future, the anomalies causing the stripe‐like patterns observed at wavefields generated by earthquakes from practically all directions (for AlpArray as well as for USArray) need to be addressed.

The paper is complemented by four appendices. Appendix A benchmarks the modeling method used, Appendix B shows the equivalence of our approach to using surface wave sensitivity kernels, Appendix C shows how the diffraction influences the group velocity (the inversion is based on phase velocity measurement), and Appendix D suggests the explanation of the observed gradiometry coefficient from the paper by Liu and Holt (2015). The Supporting Information contains animations of observed wavefield propagation both in terms of ground velocity (phase wavefronts) and energy (group velocity).

## 2 Data and Measurement

**Figure 1:**
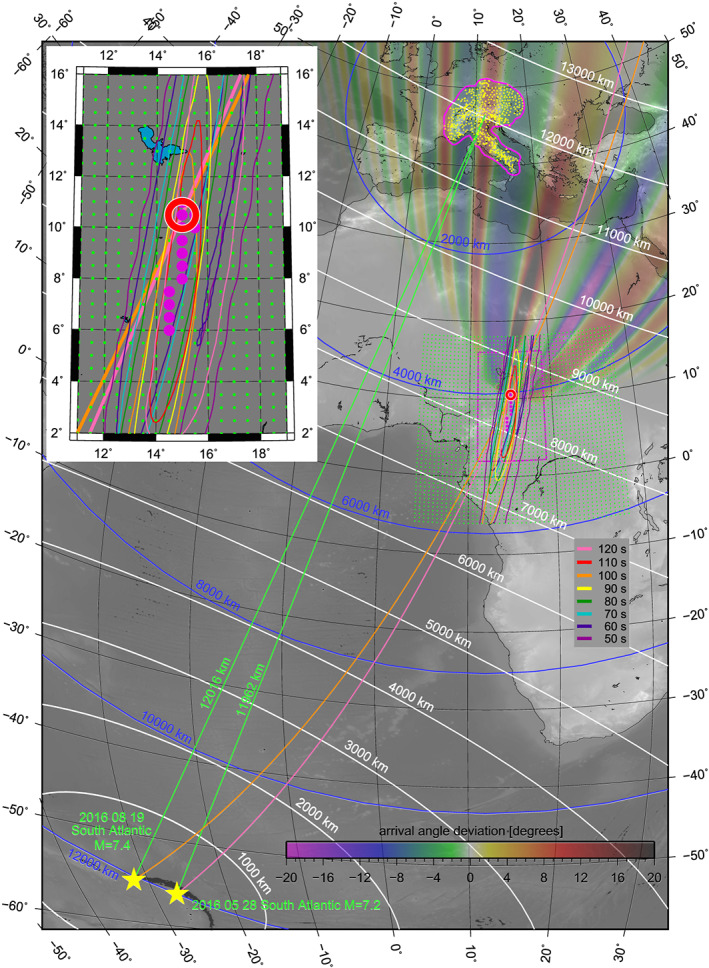
Modeled arrival angle deviations of the 100‐s Rayleigh wave propagating from the M7.4 earthquake (relative to the propagation along the great circle) and hitting the anomaly in Central Africa. The AlpArray network in Europe is shown by yellow dots. Great circle raypaths from the M7.2 and M7.4 events are shown by green lines. Distances from the source and from the AlpArray are displayed as white and blue lines, respectively. The inversion grid is shown by green dots; the confidence intervals for different periods are shown by colored lines (detail in the inset).

We selected two earthquakes, which occurred under the Southern Atlantic Ocean in 2016 (28 May and 19 August). They have similar magnitudes (7.2 and 7.4) and distances (around 12,000 km, differing by 54 km), and the epicenters are separated by 330 km (Figure 1). These two events were chosen from the 20 earthquakes investigated by Kolínský and Bokelmann (2019), since the stripe‐like patterns are clear over a broad period range and the arrival angle deviations are of high amplitude. The similarity of both events and the similarity of the observed patterns suggest that the arrival angle deviations are caused by the same structural anomaly.

**Figure 2:**
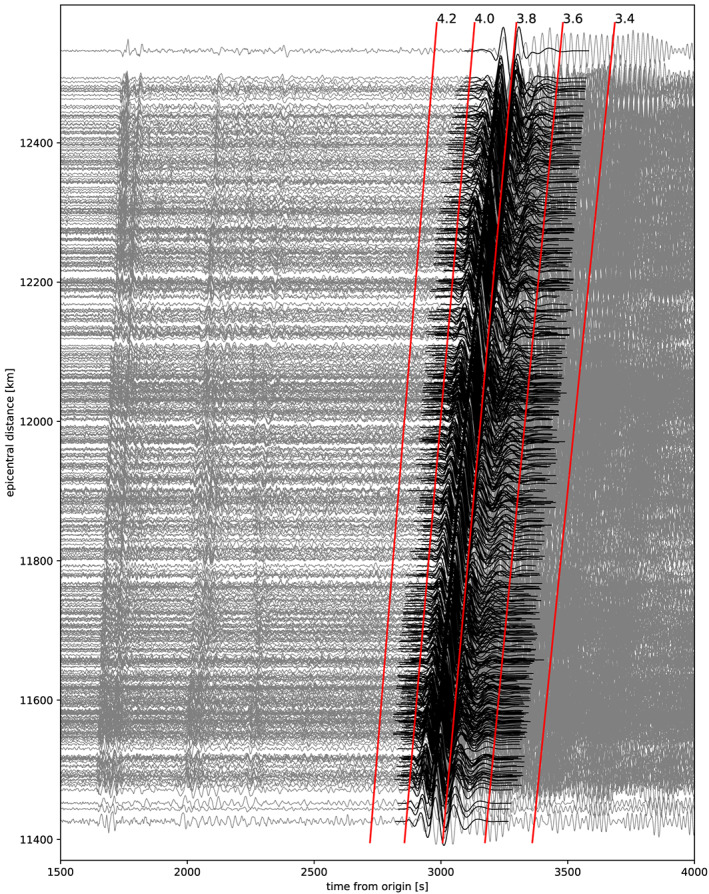
Records of the M7.4 South Atlantic Ocean earthquake (19 August 2016). Gray lines are the records filtered between 1–200 s, and black lines are the Rayleigh wave surface wavegroup filtered between 40–160 s in the frequency domain and tapered by four periods around the group velocity maximum in the time domain (data used for our measurement). Red lines show the group velocity (km/s).

We go further with exploiting the results of arrival angle deviation measurements from Kolínský and Bokelmann (2019), based on the AlpArray broadband seismic network recordings (Fuchs et al., 2015 and 2016; Hetényi et al., 2018), having 478 subarray measurements for the M7.2 and 502 subarrays for the M7.4 event (yellow triangles in Figure 1). The average subarray sizes are comparable (13.4 and 13.1 stations per subarray) for both events. As the lower limit, we used subarrays where at least five neighboring stations were found. The same rule was applied by Liu and Holt (2015), calling these “supporting stations.” However, one needs to remember that the station spacing of AlpArray is denser than was the one for USArray. The size of our subarrays is uniformly 160 km in diameter. For the purposes of the current paper, we reprocessed the data mainly to obtain the measurements for more periods. To do so, we followed the procedure of phase velocity measurement described by Kolínský et al. (2011) and the array processing developed by Kolínský et al. (2014). The overall procedure is the same as used by Kolínský and Bokelmann (2019). The magenta line in Figure 1 shows the area covered by the subarrays for the M7.4 earthquake. For the M7.2 earthquake, it differs only slightly in the western part of the AlpArray. Green lines depict the great circles between epicenters and station A291A (center of the AlpArray region). Records of the M7.4 earthquake for all 502 stations are shown in Figure 2, sorted by the epicentral distance. Gray lines are the records filtered between 1–200 s to show the complete seismograms. Black lines are the Rayleigh wave surface wavegroups filtered in the frequency domain (40–160 s) and tapered in the time domain (four periods around the group velocity maximum; see Kolínský et al., 2014 for details on the procedure). These wavegroups are the data we use in our current study. For the M7.2 event, the records and wavegroups look almost the same.

The phase velocity measurement is based on multiple filtering with bandwidths linearly decreasing from 2.9 mHz (±3.7 s) at 50‐s period to 1.4 mHz (±10.2 s) at 120‐s period. This applies for a frequency (period) content of each filtered signal over the whole record. However, as we only use the fundamental mode wavegroups tapered in the time domain (four periods in length), the actual bandwidth is much smaller, as the instantaneous frequency does not vary significantly around the maximum of the signal. The real bandwidth of our observation is then ±0.3 s at period of 50 s (0.24 mHz) and ± 0.8 s at period of 120 s (0.11 mHz). Hence, the signals used for the phase velocity determination are fairly monochromatic.

## 3 Observation

**Figure 3:**
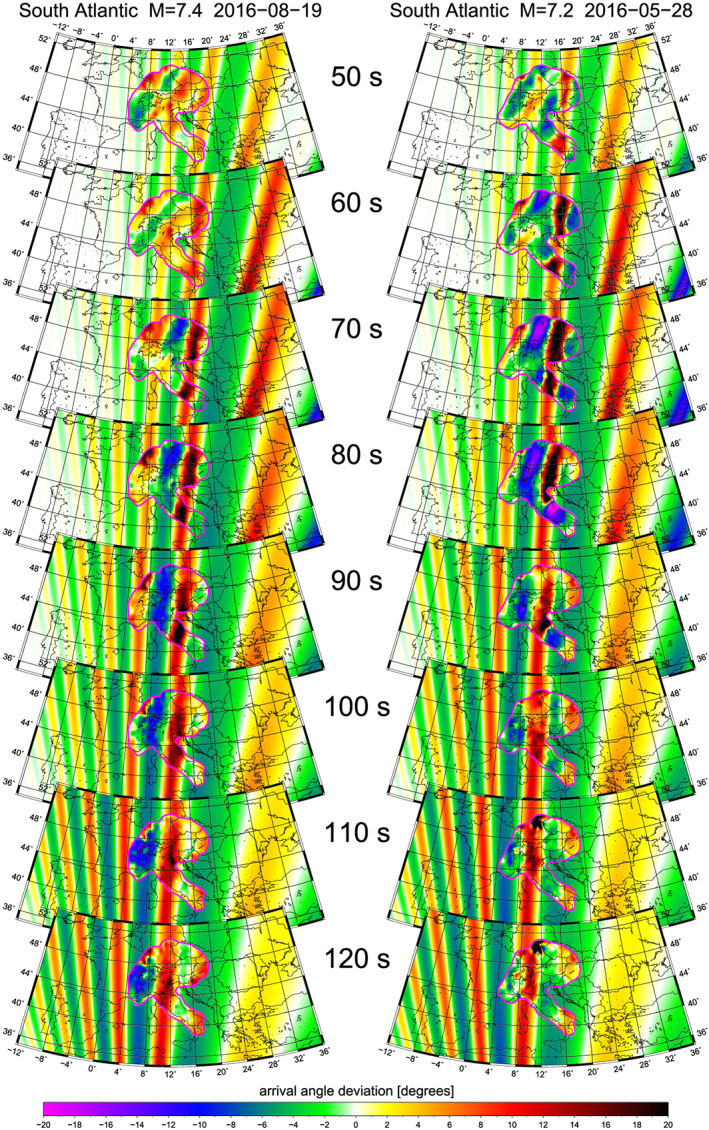
Arrival angle deviations at eight periods for the M7.4 and the M7.2 earthquakes. For comparison, the measured arrival angle deviations are shown inside the magenta‐bounded AlpArray region and the modeled arrival angle deviations outside of it. The latter are derived from the (final) best‐fitting anomaly location and parameters. Note the common westward displacement of stripes with increasing period.

In Figure 3, arrival angle deviations are plotted as the difference between measured arrival angle and geometrical great circle for eight periods between 50 and 120 s, inside the magenta‐bordered region surrounding the AlpArray. The same measurements have been presented in Figures 11 (M7.4) and S6 (M7.2) of Kolínský and Bokelmann (2019). Key observations are, on the one hand, the similarity of the patterns for the two earthquakes and, on the other hand, a lateral shift of the stripes by around 170 km. The earthquake located more to the west (M7.4) produces a pattern shifted more to the east and vice versa. It appears that the patterns for both events may be caused by the same structural anomaly, but seen from slightly different angles. Since the lateral distance of the epicenters is about twice (330 km) the lateral shift of the stripe patterns (170 km), we conclude that the position of the anomaly is roughly two times closer to the receiver array than to the epicenters, which is a consequence of the intercept theorem. This suggests a location of the anomaly in a distance of approximately 4,000 km from the AlpArray (blue circles in Figure 1).

Other key observations are that (a) there is a systematic westward shift of the pattern with period (common for both events); (b) the stripes are slightly increasing their widths for longer periods; (c) the longer the period, the more the stripe directions deviate from the great circle direction (Figure 1); and (d) the stripes are pointing east of the great circles (as seen from the network toward the source).

The period dependence of the shape and position of the stripes corresponds to the model proposed by Kolínský and Bokelmann (2019). For longer periods, the stripes are indeed increasing their widths, and they are more deviated from the great circle direction. The westward shift of longer‐period stripes suggests that the anomaly is located east of the great circles. The same is implied by the stripes pointing to the east when viewed from the network. It indicates that the anomaly is located somewhere in Central Africa.

## 4 Modeling and Inversion

N&D2000 proposed an approach based on Gaussian beams to predict the phase‐time delays *τ(x,R,T)* for a plane surface wave affected by a velocity anomaly. Kolínský and Bokelmann (2019) calculated the resulting arrival angle deviations *A(x,R,T)* as a lateral derivative of that time delay.

(1) $$A\left(x,R,T\right)=\arctan\left[c\left(T\right)\frac{\mathrm{d\tau}\left(x,R,T\right)}{\mathrm{dR}}\right]$$

In the N&D2000 formulation, the anomaly causes a Gaussian‐shaped initial time delay in an otherwise homogeneous space (2D model). The initial time delay is defined by the half width of the respective Gaussian function. We assume this half width to be equal to the half width of the causing heterogeneity, as the same is assumed in the N&D2000 paper (see also Appendix A). The velocity of the surrounding medium and the properties of the anomaly vary with period *T* and hence with depth in our modeling representing a 3D structure. Properties of the perturbed wavefield at a selected period are controlled by three parameters: the half width of the anomaly *L(T)* (full width *W(T)* = 2*L(T) in our computation), the initial time delay *τ*_max_ *(T)* of the wave right after passing the anomaly, and the phase velocity *c(T)* around the anomaly. Based on these three parameters, one can predict arrival angle deviations for any location *x* and *R* and for any period *T*. The time delay is calculated using the phase of a perturbation *Q* to a unit plane wave

(2) τx,R,T=T2πarctanIm1+QRe1+Q,

where the complex perturbation *Q* is given by

(3) Qx,R,T=ei2πτmaxTT−1ixλc,TπL2+1·exp−RL21+ixλc,TπL2.

Here, *x* is the distance of the point of interest from the anomaly measured along the great circle from the epicenter (white circles around the M7.4 earthquake in Figure 1). *R* is the lateral distance of the point of interest from the great circle which directly hits the anomaly (measured along these white circles), and the wavelength is *λ = cT*.

The N&D2000 approach has its limitations. For a given frequency, it assumes a homogeneous model everywhere around the anomaly. It is certainly a simplification of the real medium. However, we are interested only in the distortion of the wavefield, not in its absolute traveltime. The goal of the modeling is to show how the wavefield is affected when a single anomaly is assumed. We do not mean to imply that there are no other anomalies distorting the wavefield. Our goal is to show to which extent the observation can be explained by one dominant heterogeneity when all other complexities of the medium are neglected.

**Figure C2:**
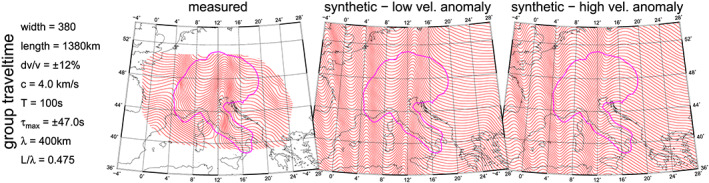
Isolines of group velocity traveltimes plotted every 10 s of propagation. Left plot shows the measurement at 100‐s period. Middle plot shows the synthetic prediction for a low‐velocity anomaly with parameters as obtained by our inversion. Right plot shows the prediction for a high‐velocity anomaly of the same size. The similarity of the two plots on the left is obvious. The right plot has the pattern “flipped.”

The time delays caused by low‐ (slow) and high‐velocity (fast) anomalies do not behave symmetrically in space (see N&D2000). Although the arrival angle deviations may look similar, especially when observing only a small portion of it (it is in general a V‐shaped pattern for both), there is a significant difference in the way the group velocity is affected by the diffraction. Comparing the observed patterns of group traveltimes with preliminary modeling results performed before the inversion and carried out for both low‐ and high‐velocity anomalies yielded that the observed pattern is caused by a low‐velocity anomaly. A pattern cast by a high‐velocity scatterer would show a significantly different shape of the traveltime contours. The comparison is given in Appendix C, Figure C2, where we show that the “U”‐shaped group traveltime contours are “flipped” for the high‐velocity anomaly. The observed shape corresponds rather to the low‐velocity anomaly.

To locate and characterize the anomaly, we perform a grid search inversion. From the preliminary results, we learnt that the phase velocity *c(T)* reached values almost identical to the PREM (Preliminary reference Earth model, Dziewonski & Anderson, 1981) for waves between 90 and 110 s. We then excluded *c(T)* from the inversion. Using Rayleigh waves measured on the vertical component, we considered v_SV_ from anisotropic PREM (omitting the water layer) and fixed *c(T)* to that model for all investigated periods. We designed a grid of locations (green dots in Figure 1) covering a large area around the distance of 4,000 km where the anomaly had been expected based on geometrical considerations (see above). The grid consists of points placed 0.5° apart between 4°E–28°E and 6°S–18°N, which is 49 × 49 = 2,401 locations in total. For each of the locations, we tested all combinations of *W* and *τ*_max_, inverting for four parameters in total: *x*, *R*, *W*, and *τ*_max_. The ranges were 100–460 km with a step of 20 km for *W* and 6–100 s with a step of 2 s for *τ*_max_, resulting in more than 2 million trials for each period *T*. For each trial, we predicted the arrival angle deviations at all stations and for both earthquakes. We computed the misfit (L1 norm) between the observed and predicted deviations for each station. We searched for the best‐fitting combination of parameters producing the smallest mean misfit (residual) calculated as the average of the misfits at all individual stations. This inversion procedure was carried out for each period *T* separately. To reach a final location common to all periods, we defined for each period *T* the confidence region as the nodes on the grid where the residual differs by less than 10% from the lowest residual for that period. Note that the values of *W* and *τ*_max_ producing the smallest residual for each location may vary among the locations. The procedure was performed for periods from 50 to 120 s with an increment of 5 s. To keep the figure legible, Figure 1 shows only eight periods with an increment of 10 s. The inset in Figure 1 shows in detail the overlap of all confidence regions. Magenta dots represent 13 points on the grid, which lie in the spatial intersection of all 15 confidence regions.

The red circle in Figure 1 represents the point with the smallest residual averaged over all periods. We consider this point, located at 10.5°N and 15.0°E, as the location of the anomaly (anomaly head). Orange and pink lines represent the great circles between the epicenters (M7.4 and M7.2, respectively) and the anomaly head. Transparent colors in Figure 1 show the predicted arrival angle deviations for a 100‐s wave propagating from the M7.4 event and interacting with the located anomaly. The orange great circle passing through the event origin and the anomaly is the axis of symmetry of that pattern. To reach the final image of the anomaly, we fixed the location to 10.5°N/15.0°E and inverted again for width *W(T)* and time delay *τ*_max_ *(T)* with refined steps of 10 km and 1 s, respectively. The period‐dependent arrival angle deviations predicted for the best‐fitting widths and time delays found for the final location are shown in the background behind the observed arrival angle deviations in Figure 3.

Our modeling approach uses a parabolic approximation of the Helmholtz equation, and further a Gaussian beam approximation, which becomes poorer for larger lateral distances from the axis of symmetry. To benchmark the approach, we used the exact Korneev and Johnson (1993) method implemented by Schneider et al. (2017). Results given in Appendix A show that for our anomaly‐stations geometry, the N&D2000 approach is applicable. Its main advantage is that it is computationally fast and hence suitable for the grid search inversion implemented.

Appendix B shows that modeling the diffraction effects (interference pattern) of the wavefield passing an anomaly is equivalent to considering sensitivity kernels for surface waves. The example is given for the 2D kernels; however, in principle, full 3D sensitivity kernels, as developed, for example, by Zhou et al. (2004), can be used to obtain the same results. Modeling one diffraction pattern for a given anomaly affecting 500 stations is, however, much faster than calculating 500 sensitivity kernels for each station separately.

To see the diffraction effects directly on waveforms and to be able to provide the same measurement on the synthetics, full‐waveform modeling would be desirable (French et al., 2013; French & Romanowicz, 2015). As the main goal of our paper is to show the principle of how the interference affects the measurement and to explain the observation by diffraction after a single distant anomaly, the simple N&D2000 approach is sufficient. Sensitivity kernels and full‐waveform modeling would increase the accuracy. To demonstrate the essence of the problem, the used method is adequate for the moment.

## 5 Results

**Figure 4:**
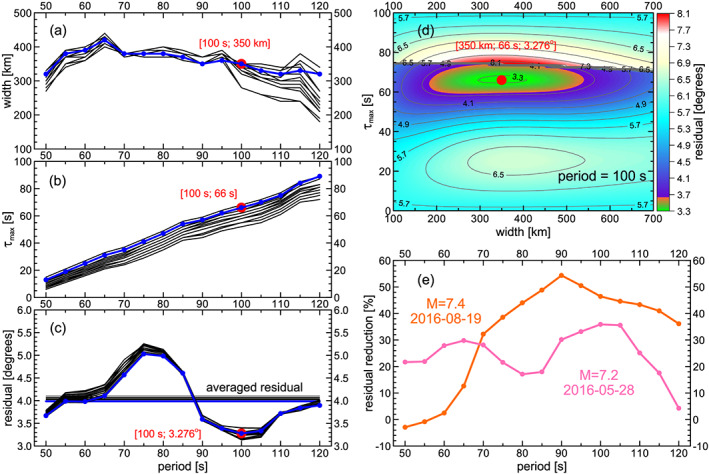
The resulting model. Anomaly widths (a), initial time delays (b), and residuals (c) for the 13 locations shown in Figure 1. Blue lines represent results for the best‐fitting location (shown by a red circle in Figures 1 and 5). (d) Misfit for the width‐*τ* *_max_* plane in model space. (e) Residual reduction (amount of deviations explained by the best‐fitting model) as a function of period, for each earthquake separately (for explanations see text).

To assess the uncertainties, we repeated the refined inversion for all 13 grid points in the intersection of the 15 confidence regions, and we present the result of the inversions for each grid point in Figure 4. Blue lines in Figures 4a–4c represent the inversion results for the best location at 10.5°N/15.0°E, and gray lines represent the other 12 locations (magenta points in Figure 1). Red dots in Figures 4a–4d highlight the values obtained at the best location for a period of 100 s.

Figure 4a shows the width of the anomaly depending on period. The values vary between 200 and 400 km, slightly decreasing with increasing period (by less than 100 km for the best solution shown by blue line). The width we are discussing represents an effective width, which is decreased by the projection of the true width onto the lateral distance *R*. However, it includes also the projection of the anomaly length onto the lateral distance *R* (along which we measure the width) if the anomaly is not hit by the wavefield exactly perpendicular to its lengthwise axis. As the anomaly length is much larger than the width (see below), the true width is thus generally smaller than the effective one. There is no systematic trade‐off between width and location, except for the longest periods, where locations to the south show smaller widths than locations in the north (not shown in Figure 4).

The initial time delay *τ*_max_ increases monotonically with period (Figure 4b), from 13 to 89 s. There is a slight trade‐off with distance: sites in the south show smaller *τ*_max_ (58‐s delay for the 100‐s wave) than sites in the north (68‐s delay for the 100‐s wave) This is due to the fact that the amplitude of the arrival angle deviation side lobes increases with distance. Hence, for more distant (south) locations, lower initial time delay is needed. There is also a slight trade‐off in the west–east direction as well: western locations require higher *τ*_max_.

After fitting, the remaining arrival angle residuals (Figure 4c) are between 3° and 5° for all locations depending on the period *T*. The residuals averaged over the periods for each location are shown by horizontal lines in Figure 4c (the best location 10.5°N/15.0°E shown again by blue color). We see that the differences between the averaged residuals for all 13 locations are quite small. This means that the other 12 locations produce only slightly worse fit to the data than the best location.

Figure 4d inspects trade‐offs between time delay and width, showing residual in the model space (at period of 100 s, for the best location 10.5°N/15.0°E). The orange color denotes 10% residual increase with respect to the optimum. While *τ*_max_ is resolved well, the width has larger uncertainty. There is little trade‐off between width and time delay. We may conclude, however, that the width is within 200 and 500 km.

Figure 3 shows a good agreement between the observed and predicted patterns for the best location. For quantitative comparison, Figure 4e shows the percentage of the arrival angle deviations which are explained by the modeling. The residual reduction is somewhat different for the two events, which can also be assessed visually from Figure 3. At shorter periods (50–60 s), there is a clear pattern visible for the M7.2 event, and the modeling fits that pattern well, which is not the case for the M7.4 event. In contrast, at the longest periods, the pattern smears out for the M7.2 event, while it is still very clear for the M7.4 event. Generally, the highest reduction is obtained in the range of 70–110 s reaching 54% at 90 s for the M7.4 event. This is the period where the arrival angle deviations are dominated by a clear pattern cast by the anomaly.

As the arrival angle deviation pattern hardly changes in N–S direction across the AlpArray, a single earthquake would give only poor constraints on the distance to the anomaly. Using two earthquakes allows for better distance estimation; however, the resulting confidence regions are still elongated in the N–S direction for all periods (Figure 1). On the other hand, the confidence regions are narrow in the W–E direction, that is, the inversion is much more sensitive to the transverse position of the anomaly than to the distance. For each period, the best‐fitting location differs in the N–S direction. Our results are based on the simplified assumption of a vertical anomaly. This appears to be justified, since we did not find any systematic trend of location with period. The difference between the averaged residuals of the best location at 10.5°N/15.0°E and the worst one (at 10.0°N/15.5°E) is only 2.8% (Figure 4c, horizontal lines). We conclude that all the 13 spots may be considered as possible anomaly locations.

The preset phase velocity of the 1D model outside of the anomaly also influences the position of the anomaly. For example, decreasing the velocity by 10% shifts the anomaly by 1° (~100 km) to the west because the stripes of the pattern are closer to the symmetry axis for lower velocities. The expected variation of the path‐averaged velocity in the region between the Central Africa and Europe is in order of units of percents. In such a case, the lateral position of the anomaly would change less than is the resolution of our grid (~50 km).

For periods shorter than 50 s, the arrival angle deviation measurement was not possible because of unclear fundamental modes. As another anomaly casts a strong pattern across the AlpArray from the SW at this period range, we could not constrain the Central Africa anomaly for shorter waves anyway. Due to our seismometer characteristics and magnitudes of the earthquakes, we were able to carry out stable measurements up to 150 s. At that period range, we still see a clear pattern for the M7.4 event. For the smaller M7.2 event, the pattern starts to be smeared from 135 s to longer waves as the number of subarrays with sufficiently low time residuals decreases due to the lower signal‐to‐noise ratio of the fundamental mode (Kolínský and Bokelmann, 2019).

## 6 Discussion

We have obtained a very good fit to the arrival angle deviations observed at the AlpArray, by assuming a single anomaly that casts a diffraction pattern to distances of several thousand kilometers. The anomaly head is found at 10.5°N/15.0°E. It has an effective width of 320–420 km, and the strength of the initial time delay increases monotonically with period from *τ*_max(*T* = 50 s)_ = 13 s to *τ*_max(*T* = 120 s)_ = 89 s.

### 6.1 Assumptions and Method

The assumption of a single anomaly may seem to be a strong one. The fit of the observations in Figure 3 speaks for itself though. Kolínský and Bokelmann (2019) have studied 20 different events, from rather different directions, and have found that essentially all of them give rise to stripe‐like patterns—different ones. The observed stripe‐like patterns also change their lateral positions with period. Thus, it is clear that these systematic arrival angle deviations cannot be caused by a local heterogeneity under the receiver array.

The arguments put forward in the observation sections indicate that the anomaly must be in Central Africa, and a formal inversion procedure has located it well in the Cameroon region. Besides the success of assuming a single anomaly for fitting the bulk of the observed pattern, we note that parts of the observations are apparently affected by other anomalies, causing a lower residual reduction at some periods. This effect is well visible at periods of 50 and 60 s for the M7.4 event (Figure 3): there are stripes of positive and negative deviations pointing in the SW direction overbeating the patterns cast by the anomaly in Central Africa. The same effect is visible for the M7.2 event as well, with lower amplitude of the SW pattern and higher amplitude of Central Africa pattern. Similarly, there is a positive stripe moving into the area of measurement from the east, visible especially for the M7.2 event from 90 s to longer periods as well as for the M7.4 event for periods of 110 and 120 s. This stripe is probably caused by an anomaly located much closer to the AlpArray region since its misalignment to the great circle direction is higher than that for the Central Africa pattern.

We point out that the aim of this study is not to explain all the observed arrival angle deviations, but rather to find out to which extent their corresponding patterns can be explained assuming a single anomaly. The other assumption of a simple geometrical shape is probably less restrictive than it may seem: diffraction effects are not very sensitive to the detailed internal structure of the causative anomaly.

We have used a simple wavefield modeling based on the approach of N&D2000. This approach involves a parabolic approximation and a Gaussian beam solution, and it is clear that it has its limits, especially when considering larger scattering angles. We test the approach by comparison with an exact solution by Korneev and Johnson (1993) implemented by Schneider et al. (2017). We find that the simple approach should be sufficiently accurate for the purposes of this paper (see Appendix A). We then profit greatly from the high computational efficiency of the N&D2000 approach, which allows performing the inversion quickly on a desktop computer. The diffraction approach in this paper can also be used to match (phase or group) velocities and, in principle, also amplitudes. It works for both body and surface waves. The effect of interference on group velocities is even much larger than that for the phase traveltimes. Observation of the energy propagating from the M7.4 earthquake is given in Appendix C and in the Supporting Information (S1 and [Link], [Link], animations of wave propagation). Appendix D then shows that the N&D2000 approach is capable to provide also other quantities used as the gradiometry observables (Liu & Holt, 2015). Being aware of the limitations and simplicity of the used method, its computational efficiency and ability to produce any property of the wavefield with unlimited spatial coverage make it a useful tool for the modeling and inversion.

### 6.2 Discussion of Results

**Figure 5:**
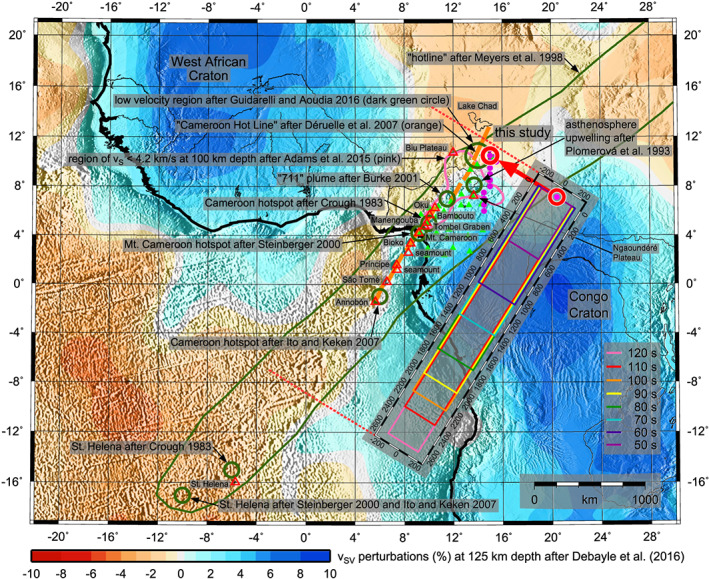
Modeled position of the anomaly head (red circle at the location of minimum mean misfit), on a geographic map with topography showing the Cameroon volcanic line (red triangles represent volcanoes). Background colors show the tomographic model by Debayle et al. (2016). Green triangles show seismic stations used by Adams et al. (2015). The inset shows the resulting lengths and widths of the found anomaly by different color for each period (see text for more information).

Figure 5 shows the position of the anomaly head for the best‐fitting set of parameters. It is located in the immediate vicinity of the CVL (Burke, 2001). That line is a 1,600 km long Y‐shaped chain of volcanoes (Halliday et al., 1990) extending both across oceanic (Meyers et al., 1998) and continental lithosphere (Elsheikh et al., 2014), which does not display any clear age progression (Guidarelli & Aoudia, 2016 and references therein). This indicates that the CVL is not just the result of a localized plume that crops out at the surface along a line due to plate motion, but that the root of these volcanoes may be rather an elongated feature in the upper mantle. An asthenospheric upwelling north of the Ngaoundéré Plateau has been discussed by Plomerová et al. (1993, Figure 5). Our location of the anomaly head closely agrees with Adams et al. (2015, Figure 5, pink region). The position also agrees with the location of a low‐velocity anomaly at the 35‐s tomography map in the ambient noise study of Guidarelli and Aoudia (2016) (dark green circle in Figure 5).

**Figure 6:**
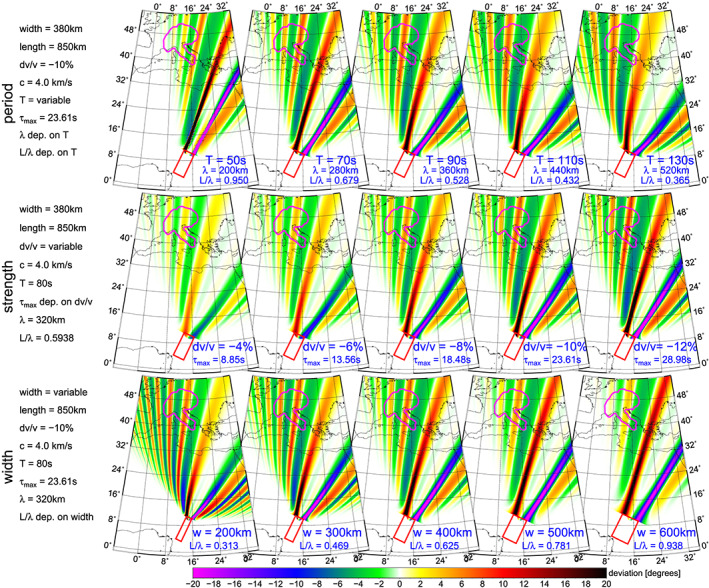
Models of arrival angle deviations for varying period, strength, and width of the anomaly (by rows of plots). In the left, the parameters are given. One of them is varied with five different values over the columns (values given in blue for each plot), while the others are kept constant. Parameters, which change depending on the variable parameter, are labeled as “dep. on …”. Their actual values are given by blue text for each plot separately.

Figure 6 explains how the diffraction pattern behaves if one model variable is changing while the others are kept constant. The three rows of maps correspond to changing the period, the strength (initial time delay), and the width of the anomaly. Parameters which are the same for all five plots in each row are given on the left side. Values which differ plot by plot are given directly for each map. The parameter *L/λ* corresponds to the same value used by N&D2000 as well as by Kolínský and Bokelmann (2019) to characterize the width of the anomaly with respect to the wavelength. We obtained the values of *L/λ* in the range given by the examples in N&D2000. Figure 6 is composed of parameters varying around the values obtained for an 80‐s wave by our inversion. We see that for longer periods (first row), the pattern significantly “opens” and the stripes are broader. Our measurement does not correspond to this for the South Atlantic Ocean earthquakes. Measurement of the patterns for earthquakes in the Aleutian Islands, Komandorskiy Islands, and two events in Ecuador (see Figures S4, S5, S15, and S17 in the paper by Kolínský and Bokelmann, 2019) shows that such a broadening does occur for these other events. For South Atlantic Ocean earthquakes, the situation is different. For longer periods, the pattern does not open that much. There needs to be something which compensates this broadening. The second row of plots in Figure 6 shows that it is the strength of the anomaly (initial time delay) which makes the pattern more “closed” and the stripes narrower. To keep the pattern closed for longer waves, the anomaly needs to appear stronger for these longer periods. This explains the general increase of the initial time delay with period (Figure 4b) obtained from the inversion.

The initial time delay *τ*_max_ is a product of anomaly strength (the velocity contrast) and length. In principle, we cannot discriminate between the two parameters. To estimate the length, however, we can guess the velocity contrast for each period. Figure 5 shows shear wave velocities (color scale, part of the global tomographic model) at 125‐km depth from Debayle et al. (2016). Rayleigh waves of 100 s have the highest sensitivity around this depth (Smith et al., 2004). Between two high‐velocity cratons, the maximum contrast reaches 12% from −2% to +10% of PREM after Debayle et al. (2016). We fixed the anomaly velocity to 98% of PREM at 100 s (3.96 km/s) for all periods and considered the cratons to have 110% of PREM across the depths. This gave us the range of contrasts from 9.1% (50 s) to 13.4% (120 s). Resulting anomaly lengths are given in Figure 5 by the kilometer‐scaled map inset with a 10‐s step in period. Even though the decreasing contrast toward shorter periods requires longer anomaly, the lengths are still dominated by heavily increasing *τ*_max_ toward long periods (Figure 4b). The length of 570 km at 50 s can be compared with the low‐velocity region around the Mt. Cameroon plume (Steinberger, 2000), “711” plume (Burke, 2001), or Cameroon hotspot (Crough, 1983). Lengths around 1,700–2,000 km at 80 to 90 s correspond to the whole CVL, (1,600 km, Halliday et al., 1990) including the Cameroon hotspot proposed by Ito and Keken (2007) or to the “Cameroon Hot Line” extending 2,000 km from the Annobón Island to Lake Chad according to Déruelle et al. (2007). Longer waves require longer anomaly, which may be compared with the tomography by Debayle et al. (2016) and to “hotline” proposed by Meyers et al. (1998), which reaches further SW to St. Helena hotspot (Crough, 1983; Ito & Keken, 2007; Steinberger, 2000).

The inset in Figure 5 shows the determined widths (320–420 km) as well (see also Figure 4a). The last row of maps in Figure 6 shows how the diffraction pattern changes, when the anomaly width is changing. Smaller width enhances the outer lobes of the pattern and smears out the main lobes close to the axis of symmetry. This behavior allowed us to reach stable estimates of the width by the inversion. In our modeling, the contrast of velocities is confined to the immediate vicinity of the anomaly. The tomographic model by Debayle et al. (2016) has much weaker velocity gradient, perhaps caused by the inevitable spatial smoothing in tomography. Tomography model by Schaeffer and Lebedev (2013) shows even lower contrasts. On the other hand, their results show a more localized low‐velocity anomaly around Mt. Cameroon for shallower depths (down to 80 km) and a more elongated body for larger depths, which corresponds to our findings.

However, the true velocity contrast of the found anomaly can be higher than what the tomography studies (Debayle et al., 2016; French et al., 2013; Schaeffer & Lebedev, 2013) suggest. If the tomography smears the effect of the anomaly in space, to obtain the same traveltimes with smaller anomaly, the contrast needs to be higher. Our estimate can hence be considered as the upper limit of the anomaly length.

The relation between the difference of shear wave velocities inside and outside of the anomaly and between the phase velocities of Rayleigh waves decreased by the anomaly with respect to phase velocities of waves propagating outside of the anomaly behaves roughly linearly. If the 1D shear wave velocity structure of the anomaly differs from the reference model outside of the anomaly by a constant shear wave velocity difference for all depths, the corresponding phase velocity curves will have the same shape. The one inside of the anomaly will have phase velocities lower by a constant difference with respect to the dispersion curve outside of the anomaly. In such a case, the initial time delay *τ*_max_ would be the same across the whole period range. If the shear wave difference increases with depth, the two dispersion curves also diverge linearly with a proportion corresponding roughly to the increase of the shear wave velocity contrast. It means that even in the extreme case, when the difference in contrast of the shear wave velocities between the depths of 75 and 150 km (50 and 100 s period) reaches several percents (we are not talking about the contrast itself, but about their difference), the difference of phase velocities at 50 and 100 s will also be in order of units of percents. This means that increasing the shear wave velocity contrast with depth can apparently increase the time delay of the longer Rayleigh waves; however, such an increase can reach units of percents at most. Such a linear trend is already accounted for in our length estimation above. Our results suggest, however, that the initial time delay is five times (500%) higher for 100‐s wave (*τ*_max(*T* = 100 s)_ = 66 s) than for 50‐s wave (*τ*_max(*T* = 50 s)_ = 13 s). The main bulk of such a difference must be accommodated by increased length of the anomaly, since the increase of shear wave velocity contrast with depth cannot account for that.

At the short‐period range, our observation shows the pattern down to 50 s. Local arrays in Cameroon (Adams et al., 2015; green triangles in Figure 5) have been able to observe the same anomaly to somewhat shorter periods, down to 30 s. This suggests that the anomaly reaches to around 50 km under the surface. We have observed the effects of the anomaly up to a period of 150 s. That period range corresponds to depths down to 200–250 km (Smith et al., 2004). The local study by Adams et al. (2015) seems to not show the anomaly at periods longer than 115 s (150 km), but this is probably due to fact that local networks with an aperture of just a few hundred kilometers cannot resolve anomalies in the deeper part of the upper mantle. Our approach illuminating the anomaly from a distance, on the other hand, is sensitive also to those depths. Closer inspection of the M7.4 event reveals some hints that the anomaly might actually extend to considerably longer periods over 160 s. Our approach is also able to identify sharp lateral velocity changes (without smoothing). This capability makes the new constraints complementary to those of tomography.

The profiles from local tomography show a mushroom‐like low‐velocity anomaly between 50‐ and 150‐km depth, with a lateral extent of 250–350 km (profiles D and E in Figure 10 of Adams et al., 2015). The width is in the range of our results. Our results suggest though that the anomaly appears as a vertical block of low‐velocity material extending at least to 200‐ to 250‐km depth, slightly narrowing downwards.

In the vicinity of our anomaly head, local tomography results show a lateral broadening up to 600 km (profile C in Figure 10 of Adams et al., 2015). Our results also hint at a wider anomaly at shallower depths and closer to the anomaly head location, but this broadening is not that significant.

### 6.3 Geodynamic Implications

We now turn our attention to the possible significance of these observations first for the CVL, then for the geodynamic processes at work. We mention several models which have been proposed to explain the CVL anomaly, and we compare these with our observation.

The tectonomagmatic CVL lies between the northern edge of the deeply rooted Congo craton and the Benue trough (Burke, 2001). It extends from the Annobón Island to Lake Chad, including 13 major volcanoes, with six of them on its oceanic part and seven on its continental part (Elsheikh et al., 2014; Lee et al., 1994; Marzoli et al., 2000; Meyers et al., 1998). Recent volcanism along the CVL displays no clear age progression (De Plaen et al., 2014; Guidarelli & Aoudia, 2016). The volume flux is small, indeed much smaller than for any of the accepted plumes (e.g., Hawaii). Yet the volcanism is active over a very long time, which renders it somewhat enigmatic. Our observations and those from tomography suggest that the upper mantle anomaly is (still) present under all the volcanoes until Annobón, and probably further to the Southwest, toward St. Helena (and the mid‐Atlantic ridge).

Several models have been proposed for the CVL. A plume origin of the magmas has been considered (e.g., Morgan, 1983), but the low volcanic volume flux and the lack of a clear age progression renders this unlikely. Alternatives have included decompression melting beneath reactivated shear zones in the lithosphere, small‐scale upper mantle convection that may advect mantle lithosphere (e.g., King & Anderson, 1998), and delamination (e.g., Fourel et al., 2013). Lateral flow of buoyant asthenosphere has also been suggested; the most prominent of these models (Ebinger & Sleep, 1998) involved lateral material transport from the Afar plume in Eastern Africa. Pérez‐Gussinyé et al. (2009) supported that study, investigating lithospheric strength via study of effective elastic plate thickness across the African continent and suggesting corridors of relatively weak lithosphere that continue across the African continent from the Afar region to Cameroon. The latter argument was, however, based in part on the lack of a clear anomaly under the CVL in early tomographic models. More recent tomographic models (Debayle et al., 2016; Schaeffer & Lebedev, 2013), as well as our study, have however begun to resolve such an anomaly in the upper mantle under the CVL. There does not seem to be a connection with a mantle plume under Afar. Our results suggest that the low‐velocity anomaly ends just south of Lake Chad. Flow from the west has also been considered (Elsheikh et al., 2014 and references therein). Connections with other volcanic region in Africa have been suggested by a number of authors, for example, for the CVL with Afar (Ebinger & Sleep, 1998; Pérez‐Gussinyé et al., 2009) and Darfur (Meyers et al., 1998). If any of these is reasonable, tomographic images (e.g., Debayle et al., 2016) would suggest Tibesti in Northern Africa and possibly Hoggar as the most likely connection, at depths between 100 and 200 km. The clear end of the anomaly to the NE in our study suggests however that the low‐velocity feature does not continue further to the NE in a substantial way.

Meyers et al. (1998) proposed a model in which heating through the transition zone drives convection cells at spatial scales on the order of 1,000 km, with alternating convective directions. They suggested that the CVL may represent the convergence of upwelling limbs from two such convection cells. It might be expected that frictional heating due to shear and heating from below would create a thermal anomaly at depth, in the transition zone. Such anomalies can be studied using receiver functions (Reusch et al., 2011). They suggested a more or less constant transition zone thickness under the CVL, similar to the global average. It is interesting to note though that at the position of our anomaly head, their receiver functions show clear thinning of the transition zone from the global average of 251 km to less than 240 km (profiles B in Figure 2 of Reusch et al., 2011). With the Clapeyron slopes given in their study, this corresponds to a thermal anomaly of around 200 K. If this represents a thermal upwelling, it would be relatively confined spatially, although to a zone of 200‐km diameter laterally. The character of this upwelling would then appear more like a localized upwelling than a cylindrical convection cell, but the location is at the edge of their study region, so the true spatial extent is difficult to tell. If this is confirmed, it would suggest an involvement of the transition zone to this upwelling. The receiver function study, however, was obviously only provided onshore. Our observation is not limited to continental upper mantle. We see asymmetric shape of the low‐velocity body: in shallower depths, it is limited to the continental part of the CVL, while with increasing depth, the anomaly gets longer, reaching the oceanic upper mantle. We can say that our image of the anomaly is more extended than the localized upwelling of Reusch et al. (2011); however, at the same time, it also does not fit into the convection cell concept of Meyers et al. (1998), which is first assumed to be of similar depth extending everywhere and which, second, continues further northeast of Lake Chad.

Other models of small‐scale convection focus on lithospheric instabilities induced by lateral variations of lithospheric thickness, for example, by the adjacent cratons (Congo Craton, West African Craton) and the continent/ocean transition (Adams et al., 2015). Thermal insulation under thicker parts of continental lithosphere may create a lateral temperature contrast and a flow from below the cratons to the CVL (King & Anderson, 1995), which has thinner lithosphere. Tomographic models would be expected to show low‐velocity zones extending across the CVL and the adjacent craton, with a shallowing under the CVL. This is not consistent with the steep tabular feature seen in our study and others. Another edge convection model considers a downwelling at the edge of thick cold lithosphere due to cooling and sinking of the surrounding mantle, while an upwelling would form under the adjacent terrain with thinner lithosphere, the CVL (Reusch et al., 2010). This would be in agreement with the linear shape of the low‐velocity anomaly and its tabular profile. This alone would not explain the extension of the volcanism to the oceanic domain though. Another small‐scale convection process was suggested by Milelli et al. (2012), who studied instabilities of viscous fluids in laboratory experiments. In the experiments, linear features appear perpendicular to continental margins, as well as branching patterns. This may explain the onshore extent, length, and orientation of the CVL, and these features may perhaps extend across the continent‐ocean transition, which would be in agreement with our results.

## 7 Conclusion

The main goal of our study had been to test whether stripe‐like arrival angle deviations that are observed in dense array data can be attributed to wave interference caused by single distant anomaly. The excellent data fit confirms that this is rather probable. We demonstrated that dense large‐scale arrays, as the AlpArray, are capable to act as antenna for remote small‐scale features. Making use of array techniques, we successfully detected, localized, and characterized a single remote anomaly by attributing the observation of stripe‐like arrival angle deviations to a wave interference effect caused by the interaction of the surface wavefield with the CVL. At the same time, we obtain important information on that interesting feature in the Earth's interior, which is visible for waves between 50 and 150 s. The low‐velocity anomaly under the CVL has a length between 570 and 2,600 km, getting longer with depth, while its width is around 370 km, narrowing for larger depths. This image of the CVL is generally similar to more recent tomographic models of the region, yet it provides additional insights due to the sharpness of the new constraints. The position of the low‐velocity anomaly near the ocean‐continent transition suggests (a) that it extends (feeds back) to the suboceanic asthenosphere and (b) that the continental edge plays an important role in the instability, if only to connect several mechanisms that generate small‐scale convection in this rather special part of the upper mantle. Most likely, several geodynamic processes enhance each other.

## Supporting information

Supporting Information S1

Movie S1

Movie S2

## Figures and Tables

**Figure A1:**
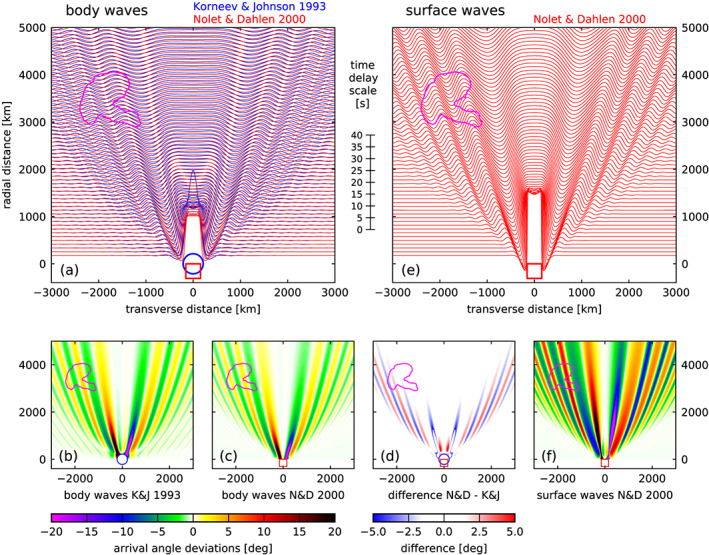
(a) Comparison of time delays from the Korneev and Johnson (1993) technique (blue) and the N&D2000 technique (red) for body waves. (b, c) Respective arrival angle deviations. (d) The difference of (b) and (c). (e) Time delays from N&D2000 technique for surface waves. (f) Arrival angle deviations for (e). AlpArray region is shown by magenta line in a position corresponding to the CVL‐AlpArray geometry. The transverse and radial ranges (km) of all six plots are the same.

**Figure B1:**
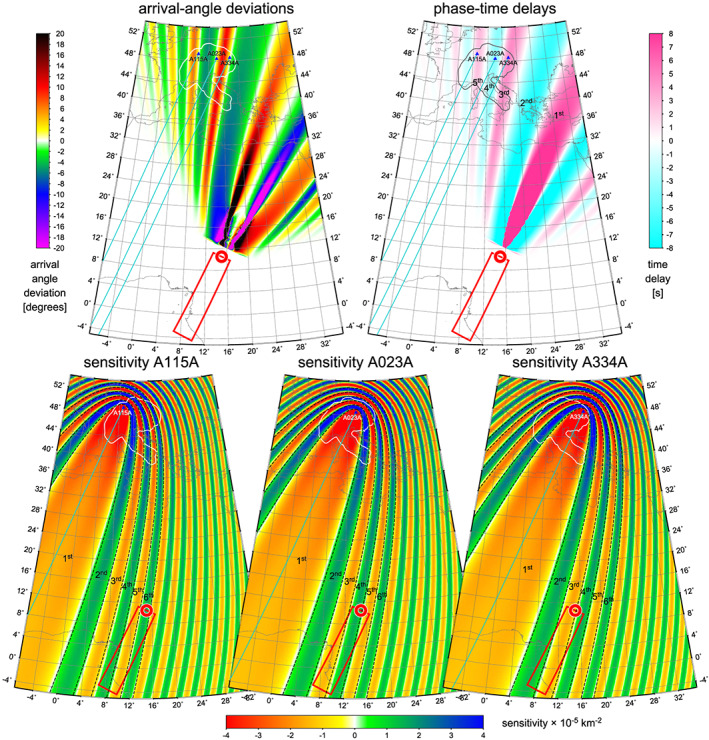
Arrival angle deviations (top left) and time delays (top right) for 80‐s surface wave traversing the low‐velocity anomaly as obtained by the inversion (red rectangle). The bottom maps show sensitivity kernels for three AlpArray stations. Fresnel zones are shown by dashed lines.

**Figure C1:**
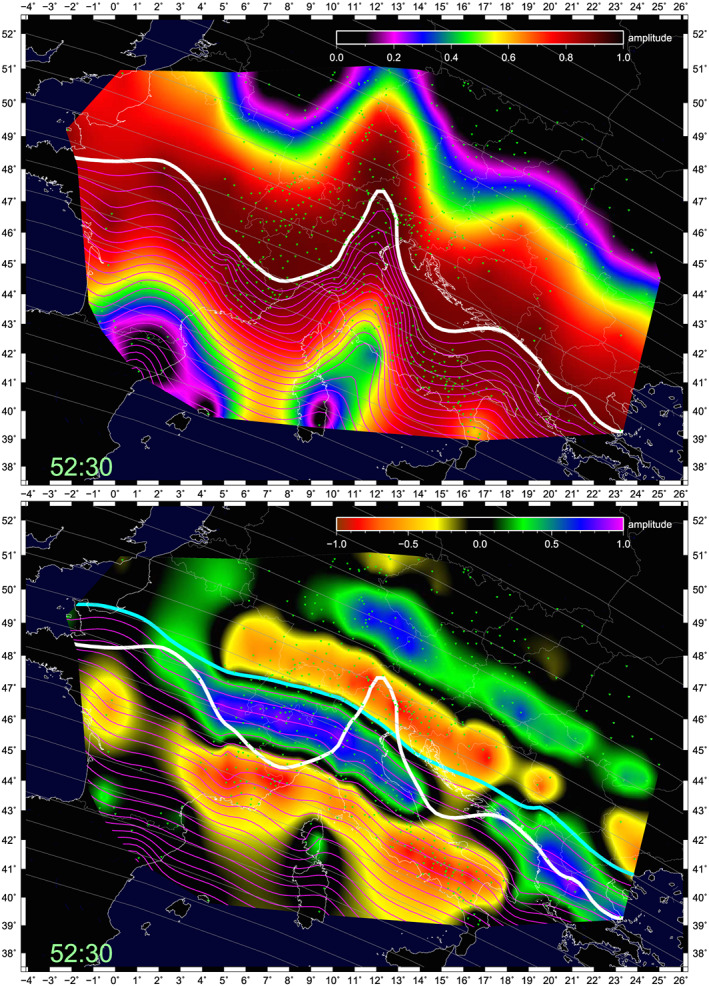
Top panel: Distribution of the energy carried by the 102‐s Rayleigh wave 52:30 (min:s) after the origin time of the M7.4 earthquake. Bottom panel: Phase amplitudes of the same wave as above. Bold white line emphasizes the energy maximum. Cyan line represents the zero crossing of the phase.

**Figure D1:**
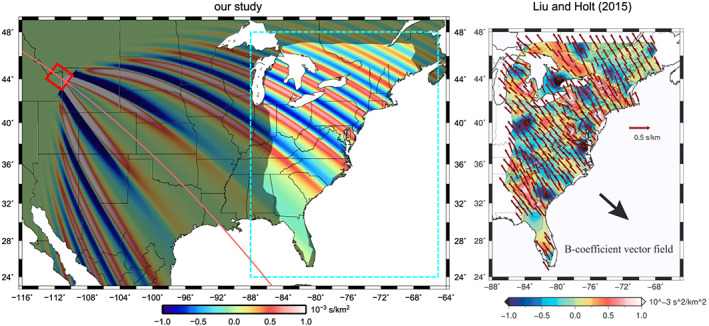
Left panel: Modeled pattern of $\vec{B}$ coefficient divergence ( $\nabla .\vec{B}$) caused by the plume under the Yellowstone hotspot (for the characteristic, see the text) for 55‐s Rayleigh wave propagating from the Honshu earthquake (M = 7.3, 25 October 2013). Right panel: Observation of the $\nabla .\vec{B}$ by Liu and Holt (2015) for the same Rayleigh wave and the same earthquake. Both color scales are the same.

## Data Availability

The data from the permanent stations can be freely accessed by the ORFEUS/EIDA repository. The data from the AlpArray temporary seismic stations (Z3) can be accessed the same way after its opening to the AlpArray Working Group (1 April 2020) and to the general public (1 April 2022).

## Appendix A Comparison With an Exact Forward Modeling Method

N&D2000 derived expressions for phase delay times of both body and surface waves. In our study, we use the approach for surface waves. It differs from that for body wave version only by the geometric term. To benchmark the Gaussian beam approach, we model the phase delays using an exact analytic solution for the interaction of a plane *P* wave with a spherical inclusion developed by Korneev and Johnson (1993), which was implemented by Schneider et al. (2017). We model a *P* wavefront passing a spherical low‐velocity anomaly. In order to retrieve comparable results to the N&D2000 approach, we avoid *P*‐to‐*S* scattering by setting the same *S* velocities inside and outside (v_s_ = 2.33 km/s) of the inclusion as well as homogeneous density of 2.7 g/cm^3^. We only reduce the *P* velocity inside the inclusion (v_p,inside_ = 2.72 km/s) compared with the surrounding (v_p,outside_ = 4.03 km/s). The analytic solution is derived for individual frequencies. For a plane *P* wave coming from below and the inclusion centered at the origin, we calculate the phase delay at several distances above the inclusion starting at the upper edge of the inclusion. The phase delay is derived by comparing the wavefields of a plane *P* wave with and without the inclusion. Time delays are computed by division of the phase delay with the angular frequency. The modeling is done in 3D for a spherical inclusion. However, we can compare the 2D and 3D situation in the central plane since in the 3D case, no energy is scattered out from that plane.

In our modeling, the N&D2000 velocity heterogeneity is represented by a boxcar causing Gaussian‐shaped time delay. The half width of that Gaussian‐shaped delay is assumed to be equal to the half width of the causing heterogeneity. In the N&D2000 paper, the shape of the heterogeneity is not defined. The distances *x* and *R* are measured along and perpendicular to the direction of wave propagation, respectively, suggesting, that a boxcar is the simplest representation of the heterogeneity keeping the *x* axis perpendicular to the *R* axis. The length of the boxcar is, together with the contrast of velocities, representing the strength in our modeling. To render the approaches of N&D2000 and Korneev and Johnson (1993) comparable, for the N&D2000 method, we used a square anomaly of the same area and the same contrast as the circular anomaly used for Korneev and Johnson (1993) solution. Hence, we talk about anomalies of the same “scattering power.” To make the comparison similar to what we observe, we use a wave with a period of 80 s hitting a circle with diameter of 350 km and a square with side of 310 km, both with a contrast of −32.5% producing an initial time delay of 36 s. The high contrast is chosen here to model the time delay similar to the observed one. The primary parameter of the N&D2000 method is the initial time delay of the wave after passing the anomaly. This initial time delay can be expressed as the product of contrast and length of the anomaly. According to our findings based on assumed contrast taken from the tomographic image (Debayle et al., 2016), the real CVL is much longer than 310 km. However, the contrast is much lower than −32.5%, resulting in similar initial time delay (tens of seconds) as in our modeling. We use a plane source and plane waves propagating in otherwise homogeneous medium for both methods.

In Figure A1, panel (a) shows the comparison of the delay times predicted by the exact analytic solution of Korneev and Johnson (1993, blue) and the N&D2000 method (red) for such an anomaly with the same “scattering power.” Both anomaly models (circle and square) are shown in the figure by respective colors. As the circular anomaly scatters the waves already as they are passing the anomaly, while the boxcar anomaly only scatters the waves when they leave the square, we aligned the center of the circular anomaly with the edge of square one. Magenta line represents the AlpArray region projected to the Cartesian coordinates (the same as in Figures 1, 3, and 6). The respective arrival angle deviations are shown at the bottom of Figure A1 in panels (b) and (c). The color scale is the same as in Figures 1, 3, and 6. The difference of arrival angle deviations from (b) and (c) is given in panel (d). We see an overall good agreement of the two techniques. The delay times show patterns with flat time delay curves for emergent angles in the ±10° range. For larger angles, the delay time curves show strong modulations, with a delay time minimum and maximum at emergent angles of around ±15° and ±20°, respectively, giving the whole pattern the appearance of a “V shape.” They start to differ for larger lateral distances, and there is a difference right at the central region behind the anomaly itself. The N&D2000 technique predicts smaller time delays at the central region; however, after recalculating to the arrival angle deviations, we see a good match. At the distances considered for the AlpArray, the amplitude of the time delays and the arrival angle deviations are almost the same. Maxima and minima of the time delays are slightly closer to the center for the N&D2000. Figure A1, panel (e), shows the prediction by N&D2000 for surface waves of 80 s (the same period and velocity and hence also wavelength as on the left panel (a) in case of body waves) passing exactly the same anomaly as in panel (a). This plot shows an example of the patterns used for the inversion. Arrival angle deviations for this surface wave model are shown in panel (f). We see that for surface waves, the amplitude of the time delays (and hence also of arrival angle deviations) is much larger, indicating that the anomaly is seen for larger distances. In other words, the wavefield is not healed that much as in the case of body waves. The whole “V‐shaped” pattern is also a bit more closed than in the case of body waves.

The N&D2000 method uses the parabolic approximation of the Helmholtz equation and further a Gaussian beam approximation. The approximations become poor for larger lateral distances. According to N&D2000, the approximation is accurate to about θ = 15°, where θ is the emergent angle between wave vector **k** and the positive *x* axis for a plane wave propagating in *x* direction. In fact, for the considered geometry, we apply N&D2000 for θ ~ 30°, which we validate with the presented comparison with an exact solution of the Helmholtz equation. In terms of calculation expenses, the N&D2000 approach is much faster since it predicts the time delay for any point when only the coordinates of that point are given in a single expression. As opposed to that, the exact analytic solution by Korneev and Johnson (1993) is making use of an infinite spherical harmonic expansion of the function space, which has to be truncated at a certain number. The larger the anomaly is and the higher the frequencies of the incident wavefield are, the more elements of the infinite series have to be taken into account. This makes the calculation expensive for large anomalies and/or high frequencies.

## Appendix B Equivalence to Sensitivity Kernels

We predict interference patterns cast by a velocity anomaly, and we show how the associated arrival angle deviations change with position in space (over the network of stations). For fixed source and anomaly position, we predict alternating stripes over the AlpArray. This approach is in essence equivalent to considering sensitivity kernels for surface waves. Sensitivity kernels predict the effect of varying anomalies (position, size) for fixed source and receiver positions. To demonstrate this, we adopted the 2D sensitivity kernels obtained by Zhou et al. (2004). They derived full 3D kernels showing that when considering the forward scattering approximation and neglecting mode coupling effects, these 3D kernels for structural parameters (shear and longitudinal velocity, density) can be simplified to 2D kernels for surface wave phase velocities. As the primary output of the N&D2000 equation is the phase‐time delay, we also use the kernels predicting phase delays to indicate the equivalence. In explicit form, these 2D sensitivity kernels predicting the phase delay *δϕ* caused by the perturbation *δc/c* of the reference phase velocity *c* at position *x* for single frequency *ω* are given in Yang and Forsyth (2006) as

(B1) Kϕcxω=Imk2e−ikx″−kΔx+π/42πkx″,

where *k* is wavenumber, *x*″ is the distance from the scatterer to the receiver and Δx is the differential distance between the scatterer and receiver projected to the direct raypath (see Figure 1 in Yang and Forsyth, 2006). The kernels are calculated for a plane wave. We mapped them to the sphere using the same procedure as for the arrival angle deviations. The use of the kernels predicting the phase delay *δϕ* follows

(B2) $$\delta \phi =\iint_{\Omega }K_{\phi }^{c}\left(x,\omega \right)\left(\mathrm{\delta c}/c\right){\mathrm{dx}}^{2}.$$

At each spot, we combine the sensitivity and the velocity perturbation and we integrate these products over the Earth surface.

The upper left‐hand‐side map in Figure B1 shows the arrival angle deviations for the Cameroon volcanic line (CVL) size and position as obtained from the inversion for Rayleigh waves with a period of 80 s for the South Atlantic M7.4 earthquake. This pattern was also shown in the background of the respective period map in Figure 3 (left column, fourth row). The upper right map in Figure B1 shows the corresponding phase‐time delays in seconds. The arrival angle deviations on the left are calculated as a lateral derivative of these time delays (see Equation 1). Hence, regions of zero arrival angle deviations (white stripes in the left‐hand‐side map) correspond to maxima and minima of the phase‐time delays (dark pink and dark cyan regions in the right‐hand‐side map). We selected three AlpArray stations so that they lie at these stripes where the arrival angle deviations are zero and the phase‐time delays are extreme. We calculated the sensitivity kernels for these three stations (see the bottom maps in Figure B1). For the sensitivity kernels, we use a color scale with negative values depicted in red. Negative sensitivity means that the wave is slowed down by a low‐velocity anomaly, since in this case, both terms in the integral of Equation B.2 are negative, resulting in a positive value of the phase delay. We see that for the three stations, the CVL anomaly (red rectangle) is situated in different branches of the positive and negative stripes of the sensitivity kernels. This corresponds exactly to the manner in which the CVL is casting the positive and negative stripes of phase‐time delays over the AlpArray. For station A334A, the CVL anomaly lies in the “slow” third Fresnel zone (orange) slowing down the wave, which corresponds to the fact that A334A lies at the maximum of the phase‐time delays (dark pink stripe in the upper map). Sensitivity for A023A is dominated by the “fast” (green) fourth Fresnel zone, although both “slow” third and fifth zones contribute a bit as well. For A115A, the CVL anomaly reaches already up to the sixth Fresnel zone; however, the “slow” fifth one (which is the third “slow” zone counting only the odd ones) contributes the most.

Also note that while in Yang and Forsyth (2006), the sensitivities are smoothed by a 50‐km Gaussian window, we do not smooth the sensitivities at all to keep the higher Fresnel zone contributions visible. Modeling of both the sensitivity kernels and the diffraction patterns is done for a single frequency. Also note that while the Fresnel zones in Figure B1 (dashed lines) are exact ellipses plotted on the sphere, the sensitivity kernels are recalculated from the plane. This is why they do not match the exact Fresnel zones perfectly. Sensitivities are asymmetric in sign: the minimum just in front of the station (first Fresnel zone) reaches the value of −21 × 10^−5^ km^−2^ while the maximum (second Fresnel zone, close to the station) is only +11 × 10^−5^ km^−2^. We, however, keep the color scale saturated between ±4 × 10^−5^ km^−2^ to allow the visibility of the higher Fresnel zones with decreasing sensitivities.

The comparison between interference patterns and sensitivity kernels also shows that it is important to consider higher Fresnel zones in tomography. We observe the effects of the sixth Fresnel zone at the stations in the western part of AlpArray.

## Appendix C Group Velocities

Our study is based on measuring the phase velocities, better say, their arrival angles. We used the array approach, which allows for determination of both the velocity and its direction. In addition, it also allows for data quality assessment as described in the Appendix by Kolínský and Bokelmann (2019). Every station in every subarray has its residual for every period, and hence, outliers with significantly higher residuals can be removed. This makes the arrival angle calculation precise enough to observe and invert the stripe‐like patterns discussed in the current paper. As shown by N&D2000, group velocities are affected by the interference of diffracted waves as well. The important point to note is that group velocity is, in general, affected much more. The time delay caused by the same anomaly observed at the same location is higher for the group velocity, and this effect is more pronounced for larger lateral distances *R*. This is demonstrated in Figures 6 and 8 in the N&D2000 paper. It manifests itself both by the healing of the group time delays being smaller right along the central raypaths (*R* = 0, epicenter–anomaly) as well as by the side lobes being more pronounced off the central raypath. While low healing along the central raypath could be a good news for the tomography based on the ray approximation, anomalies off the direct raypaths introduce much larger time delays for the group velocity measurement than for the phase.

To complement our observation of phase velocities, we also performed the group velocity measurement. There are qualitative differences between the two measurements. First, the group velocity can be measured using a single station. This looks like an advantage. Any station in the region of interest can be used without considering subarrays and their geometry of neighboring stations. It is enough to simply evaluate the group velocity for each station separately and plot the value in the map. However, there are two main disadvantages. The first one is that the estimation of the group velocity is less precise than that for the phase. Group velocity measurement uses the envelope of the record at a given period. As the envelope is a slowly varying function with time, the time of its maximum is determined with lower accuracy than the differential time of the phase when correlating two records at neighboring stations. In case of the phase velocity, we can reach even the subsample precision (see e.g., Brokešová and Málek, 2018). This is not possible for the group velocity. The second disadvantage is that when using single stations, the data quality assessment is difficult. It cannot be performed using the residuals as in the case of subarrays. It can still be done using, for example, signal‐to‐noise ratio. However, timing issues cannot be addressed. While, in principle, the group velocity can be used for the inversion as well, because of these issues, we based the study on the precise phase velocity measurement. To demonstrate how the group velocity distribution looks like, nevertheless, we plot the pattern for the period of 102 s in Figure C1, upper panel, for the M7.4 South Atlantic Ocean earthquake. We use all the stations of the AlpArray project as in the case of the phase velocity subarray measurement, and we added 92 stations distributed around Europe (green triangles). For these additional stations, a rough data quality assessment was performed based on visual inspection and signal‐to‐noise ratio. Group velocity was estimated using the method described by Kolínský and Brokešová (2007) based on multiple filtering. This procedure is anyway part of the phase velocity calculation, as mentioned in Kolínský et al. (2011). Figure C1, upper panel, shows a snapshot in the time 52 min and 30 s after the origin time. Color denotes the distribution of energy over the network (normalized to 1 at its maximum), and white line emphasizes this maximum. Magenta lines are the positions of the energy maxima in previous times plotted for every 10 s (group traveltime contours). Gray thin lines in the background show the great circle wavefronts. The figure is taken from an animation showing the propagation of the 102‐s wave energy over the network. The animation is given as Supporting Information S1 to this paper. The time runs from 46:00 (min:s) to 59:00. It shows how the consistent pattern of advanced and delayed portions of the energy is moving across the network.

The comparison of the phase‐wavefront propagation is shown in Figure C1, lower panel. Color scale now refers to positive and negative amplitudes of the 102‐s wave directly. Arbitrarily chosen zero‐crossing point of the wavefield is emphasized by the cyan line. Again, the magenta contours are the positions of the phase wavefront in previous times plotted for every 10 s. White line showing the energy maximum is the same as in the upper plot. The lower panel is also taken from the animation given as Supporting Information S2 to this paper. We see that the phase‐wavefront distortion is in order of units of seconds with respect to the circular wavefronts, while the distortion of the envelope is an order of magnitude larger (tens of seconds).

To demonstrate the asymmetrical effects caused by low‐ and high‐velocity anomalies (N&D2000), Figure C2 shows the predicted group traveltimes for 100‐s wave affected by both negative and positive scatterers. We see that while a low‐velocity anomaly produces broad stripes of apparently low group velocities and narrow stripes of apparently high group velocities, in the case of the high‐velocity anomaly, this pattern is “flipped”—the high apparent group velocities show broader stripes. The comparison with the observation supports the initial assumption of our study that the particular observed pattern is caused by a low‐velocity scatterer. The dimension and strength of the anomaly used for the synthetic prediction in Figure C2 were taken from our inversion. Note that the initial time delay of ±47 s used to model the effect at 100‐s wavegroup velocity corresponds to the delay obtained for a period of 80 s when inverting the arrival angles based on the phase velocity measurement. It corresponds to the group velocities being sensitive to shallower depths then phase velocities at the same period; in other words, the same depth affecting phase velocity at the 80‐s period roughly corresponds to the depth affecting the group velocity at the 100‐s period. We used this initial time delay to qualitatively match the group traveltime observation shown in the left panel of Figure C2. As the resulted initial time delay strongly varies with period, using the time delay *τ*_max_ = 66 s, as obtained for the phase velocity at 100 s, would yield a pattern of group traveltimes shifted laterally in space with respect to observation. Looking at the group velocities thus additionally supports the conclusion that the change of the initial time delay is caused by the anomaly strength varying with depth.

## Appendix D The Yellowstone Plume

As mentioned in section 1, stripe‐like patterns of various observables were measured using the USArray data (Foster et al., 2014; Liang & Langston, 2009; Lin et al., 2012; Liu & Holt, 2015; Pollitz, 2008). Here we look more closely at the study of Liu and Holt (2015). In Figure 14a, they show the results of gradiometry coefficient $\vec{B}$ as measured using Rayleigh waves of 55‐s period propagating from the Honshu earthquake (M = 7.3, 25 October 2013, 37.156°N, 144.661°E). Vector $\vec{B}$ is a negative slowness, meaning, it is −∇τ (local gradient of the phase traveltime). The color map in the latter figure shows $\nabla .\vec{B}$ (divergence of $\vec{B}$) which is then −∇·∇τ = −∇^2^τ = −Δτ (Laplacian of the traveltime). The direct output of our modeling is the phase traveltime. As we can calculate this quantity for arbitrary point in the space, by spatial differentiating, we can easily provide any of the gradiometry quantities, including $\nabla .\vec{B}$. For curiosity, and as a teaser for what can be done in the future, we model the $\nabla .\vec{B}$ of the wavefield distorted after passing the Yellowstone plume for the same earthquake and the same 55‐s Rayleigh wave as in Liu and Holt (2015). To model the effect, we needed the position and size of the Yellowstone plume, as well as the phase velocity of 55‐s Rayleigh waves in the Eastern United States. As shown by Smith et al. (2004), 55‐s Rayleigh wave phase velocity has its peak sensitivity for depths around 75 km. Waite et al. (2006) calculated the v_s_ structure beneath the Yellowstone hotspot. Their Figure 6 shows that the SE edge of the low‐velocity anomaly is centered at 43.7°N and 110.5°W for the depth of 90 km, which is the closest to what we needed. We took this location as the “anomaly head,” as the waves are propagating from NW to SE (see the pink great circle line connecting the Honshu earthquake epicenter and that Yellowstone plume “head” in Figure D1). As the tomography results by Waite et al. (2006) produced smooth image of the Yellowstone plume, the anomaly width cannot be accurately determined. For our purposes, we set it to 180 km (the size of the slowest patch in Figure 6 by Waite et al., 2006). The phase velocity of 4.1 km/s is taken from Pollitz and Mooney (2016, see Figure S2 in the Supporting Information to their paper). The only unknown parameter is then the strength of the anomaly, which in the case of its length being around 150–200 km (see again the results of Waite et al., 2006 and the red rectangle in Figure D1) needs to be, however, much larger than resulted from the tomography by the latter‐mentioned study. As the strength of the anomaly practically only influences the strength of the observed stripes (of any quantity) and not that much their spatial distribution, we arbitrarily decided for a peak‐to‐peak strength of 20% to produce a pattern similar to the one observed. The model is shown in Figure D1 (left panel) compared with the original measurement from Figure 14a by Liu and Holt (2015) in the right panel of Figure D1. Both maps are in scale, and both color scales are the same. Our model has dimmed brightness outside of the measurement area of Liu and Holt (2015) to emphasize the results in the area of interest (Eastern United States). We see the same number of positive and negative stripes of the same amplitude pointing in the same direction. Liu and Holt (2015) also gave a measurement of the back azimuth variations. We, however, choose the $\nabla .\vec{B}$ for the comparison for two reasons. First, Liu and Holt (2015) gave continuous stripes of $\nabla .\vec{B}$ over the whole area of interest, and hence, the resemblance of their results to the diffraction pattern is striking. Second, we want to show that our modeling is capable to provide any (gradiometry) quantity. The significance of $\nabla .\vec{B}$ over the back azimuth variations or over the phase traveltimes themselves is that as $\nabla .\vec{B}$ can be understood as a second spatial derivative of the phase traveltimes, it enhances the amplitude of outer lobes (stripes). The phase traveltime itself has decreasing variations in lateral direction from the axis of symmetry of the diffraction pattern. As these stripes are varying over shorter distances, its derivative in this direction makes the outer stripes stronger. The second derivative strengthens them even more. It can be seen in our model: the third to fifth positive $\nabla .\vec{B}$ stripe in the area of interest (Eastern United States) is more pronounced (white ridges) than the first and second positive stripe closer to the axis of symmetry. In this example, our intention is not to introduce any conclusion about the structure of the Yellowstone plume in the upper mantle, neither to claim that all the variations of $\nabla .\vec{B}$ given by Liu and Holt (2015) can be explained by such a simple model. We show it here to point out what could be the effect of such an anomaly (upper mantle plume) on the surface wavefield and that it could affect the wavefield in a similar way as observed.
