# Supplementary material for: Surface Wave Diffraction Pattern Recorded on AlpArray: Cameroon Volcanic Line Case Study
Source: J Geophys Res Solid Earth. 2020 Jul 21;125(7):e2019JB019102. doi: 10.1029/2019JB019102 (PMC7507139; doi:10.1029/2019JB019102)
Supplement: Supplementary file 1 — Supporting Information S1 [file JGRB-125-e2019JB019102-s003.pdf]

Journal of Geophysical Research – Solid Earth

Supporting Information for

Surface wave diffraction pattern recorded on AlpArray: Cameroon Volcanic Line case study

Petr Kolínský[1], Felix M. Schneider[1,2], Götz Bokelmann[1]

(1) Department of Meteorology and Geophysics, University of Vienna, Vienna, Austria

(2) Section "Seismology", Helmholtz Centre Potsdam – German Research Centre for Geosciences (GFZ), Potsdam, Germany

Contents of this file

Description to Animations S1 and S2

Additional Supporting Information (Files uploaded separately)

Animations S1 and S2

Rayleigh wave propagation across the AlpArray network

Online-only Supporting Information consists of two animations. Both show the propagation of the 102 s Rayleigh wave (vertical component) from the M7.4 earthquake which occurred under the South Atlantic Ocean on August 19, 2016, see the "Data and measurement" section in the text. They start 46:00 minutes after the origin time and proceed up to the time of 59:00. They are accelerated with a factor of 30, meaning, every second of the movie represents 30 s of the real time. Animation S1 shows the propagation of the energy carried by that 102 s wave and Animation S2 shows the amplitude of the same wave. White solid line in both animations represents the envelope maximum. Cyan line in the Animation S2 shows the zero crossing of the phase representing the wavefront. Animations were created using 594 stations of the AlpArray project as well as permanent stations across Europe. After resampling the data to 1 sps, a map of amplitudes as well as of envelopes of the narrow-band filtered quasiharmonic signal at 102 s was plotted for every second. A set of 781 (13 minutes) maps was then used to compile the movies.
